# Supplementary material for: Polymorphism of VRTN Gene g.20311_20312ins291 Was Associated with the Number of Ribs, Carcass Diagonal Length and Cannon Bone Circumference in Suhuai Pigs
Source: Animals (Basel). 2020 Mar 13;10(3):484. doi: 10.3390/ani10030484 (PMC7142862; doi:10.3390/ani10030484)
Supplement: Supplementary file 1 [file animals-10-00484-s001.pdf]

animals

Supplementary

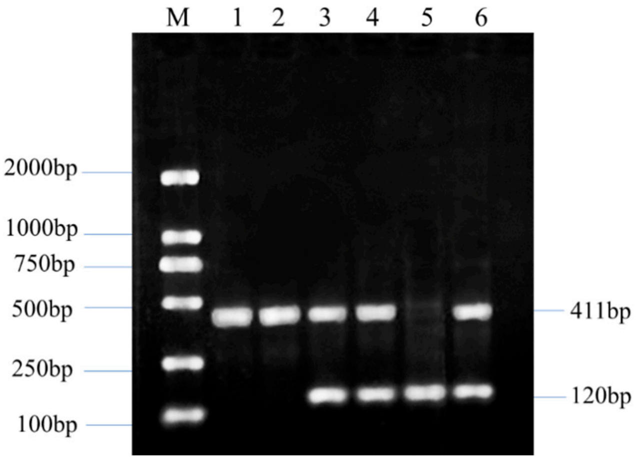

Supplementary Figure 1. Genotyping for the *VRTN* gene G.20311_20312ins291 mutations. Lanes 1 to 6 showed PCR products. M: 2000bp DNA Marker; 1 and 2: ins/ins genotype; 3,4 and 6: ins/del genotype; 5: del/del genotype.

Table S1. Correlation analysis between batch/sex/age and RIB/carcass traits of Suhuai fattening pigs.

Traits	p_{batch}	p_{sex}	p_{age}
RIB	0.3341	0.4585	0.4030
CSL	0.0046	0.0493	0.0030
CDL	0.0000	0.0034	0.0014
CWT	0.0000	0.0000	0.0012

Note: RIB, number of ribs; CSL, carcass straight length; CDL, carcass diagonal length; CWT, carcass weight; p_{batch}, p_{sex} and p_{age} represent the p-value of the correlation between RIB/carcass traits and batch/sex/age of Suhuai fattening pigs, respectively.

Table S2. Correlation analysis between batch/sex/age and body size traits of Suhuai gilts.

Traits	p_{batch}	p_{sex}	p_{age}
CC	0.0000	0.0000	0.7942
AC	0.0000	0.0029	0.5820
CBC	0.0000	0.0000	0.5415
RC	0.0000	0.0000	0.1380
BL	0.0000	0.0000	0.9559
BW	0.0000	0.0018	0.3504

Note: CC, chest circumference; AC, abdominal circumference; CBC, cannon bone circumference; RC, rump circumference; BL, body length; BW, body weight; p_{batch}, p_{sex} and p_{age} represent the p-value of the correlation between body size traits and batch/sex/age of Suhuai gilts, respectively.
